# Residence in HUD Housing Associated With Greater Benefit From HCBS Services for Medicaid Enrollees in Pennsylvania

**DOI:** 10.1093/geroni/igab046.1839

**Published:** 2021-12-17

**Authors:** Damian Da Costa, Howard Degenholtz

**Affiliations:** 1 University of Pittsburgh, Pittsburgh, Pennsylvania, United States; 2 University of Pennsylvaniz, Pittsburgh, Pennsylvania, United States

## Abstract

State Medicaid programs seek to shift the delivery of long-term care services away from institutional settings and toward community-based settings by expanding access to home-and-community-based services (HCBS). HCBS are hypothesized to prevent or delay the need for protracted nursing home stays. This study explores the question of which types of community residence maximize this protective effect of HCBS. We used a probabilistic matching technique to identify whether waiver-eligible Medicaid enrollees were likely to reside in project-based HUD housing in 2013. We applied multinomial logistic regression to observe the risk of long-stay nursing home admission (>100 days) relative to persistent community residence in the subsequent four years. Our model controlled for age, race, gender, urban status, and receipt of home-and-community based services. Our predictor of interest was the interaction between receipt of home and community based services (HCBS) and residence in HUD housing. The eligible baseline population included 152,632 community-residing Pennsylvania Medicaid enrollees in 2013. The analytic sample excluded individuals who died during 2013 or who were no longer waiver-eligible after 2013. Residence in HUD project-based housing while receiving HCBS is independently associated with a 27% percent reduction in risk of long-stay nursing home admission (p = .01) when controlling for individual-level demographics. No significant association was observed between the predictor of interest and risk of death during the follow-up period, suggesting that this finding is not likely confounded by individual health status. Further research should test whether this association is causal and specify possible mechanisms.

